# Traditional health practitioners’ perceptions, herbal treatment and management of HIV and related opportunistic infections

**DOI:** 10.1186/1746-4269-10-77

**Published:** 2014-12-05

**Authors:** Denver Davids, Tarryn Blouws, Oluwaseyi Aboyade, Diana Gibson, Joop T De Jong, Charlotte Van’t Klooster, Gail Hughes

**Affiliations:** Department of Anthropology and Sociology, University of the Western Cape, Bellville, South Africa; South African Herbal Science and Medicine Institute, University of the Western Cape, Bellville, South Africa; Amsterdam Institute for Social Science Research (AISSR), University of Amsterdam, Amsterdam, The Netherlands

**Keywords:** HIV, TB, Traditional health practitioners, Medicinal plants

## Abstract

**Background:**

In South Africa, traditional health practitioners’ (THPs) explanatory frameworks concerning illness aetiologies are much researched. However there is a gap in the literature on how THPs understand HIV-related opportunistic infections (OIs), i.e. tuberculosis, candidiasis and herpes zoster. This study aimed to comprehend THPs’ understandings of the aforementioned; to ascertain and better understand the treatment methods used by THPs for HIV and OIs, while also contributing to the documentation of South African medicinal plants for future conservation.

**Methods:**

The study was conducted in two locations: Strand, Western Cape where THPs are trained and Mpoza village, Mount Frere, Eastern Cape from where medicinal plants are ordered or collected. Semi-structured interviews were conducted with 53 THPs of whom 36 were diviners (*amagrirha: isangoma*) and 17 herbalists (*inyanga*). THPs were selected through a non-probability “snowball” method. Data were analysed using a thematic content analysis approach. An ethnobotanical survey was conducted and plants used to manage HIV and OIs were collected. A complete set of voucher specimens was deposited at the University of the Western Cape Herbarium for identification. Plant names were checked and updated with Kew’s online website http://www.theplantlist.org.

**Results:**

THPs conceptualise the aetiology of HIV and OIs at two related levels. The first involves the immediate manifestation of the illness/condition because of a viral infection in the blood (HIV), the presence of bacteria in the lungs (tuberculosis), or weakened state of the body making it susceptible to OIs. The presence of OIs is indicative of the probable presence of HIV. The second level of causation affects the first, which includes pollution, changes in cultural sexual norms, witchcraft, environmental factors, and lack of adherence to ancestral rituals. THPs reported using 17 plants belonging to 12 families. Remedies included mixes of up to five plants.

**Conclusion:**

This study explored the THPs’ perspectives on HIV and commonly associated OIs and their herbal treatment methods. THPs generally rely on biomedical diagnosis before treating a client. They also seek guidance from the ancestors for a particular diagnosis, the plants to use for a specific treatment, when to harvest, and how to administer herbal remedies.

**Electronic supplementary material:**

The online version of this article (doi:10.1186/1746-4269-10-77) contains supplementary material, which is available to authorized users.

## Background

South Africa has a high prevalence of HIV/AIDS and a plethora of related opportunistic infections (OIs) including tuberculosis (TB), herpes zoster and candidiasis. About 6.4 million South Africans were living with HIV in 2012 [1]. The incidence of TB in the country was approximately 400 000 in 2010 [2]. HIV and TB are also preeminent in South Africa in relation to morbidity and mortality [3]. The pervasiveness of these diseases has heavily impacted the country’s health-care service delivery system. Although the roll-out of antiretroviral therapies has increased annually, approximately 2.7 million South Africans are still in need of Anti-retroviral (ARV) treatment [4]. This situation has led to a search for possible alternative and/or complementary treatment options. Moreover, South Africa, like other African countries, also relies on the use of its traditional healing systems [5–7]. Traditional medicine (TM) is being formalised and is increasingly seen as part of the national health resources of South Africa [8].

TM is widely used for addressing minor infections, or when ARV’s cannot be taken, for example due to drug resistance [9]. Several studies in South Africa show that TM co-exists with allopathic medicine and health-care services and people are likely to combine them [10–12]. According to Van Niekerk [8] and Bateman [13], many South Africans first attempt to self-diagnose and treat or seek help from traditional health practitioners (THPs) before consulting formal health-care services. Many also turn to TM as a way to prevent or relieve the side effects of ARV medication [14]. There is also demand for TM which increases immunity or improves overall health and well-being. Such demand is strengthened by the availability of inexpensive alternatives to allopathic medication in local pharmacopeia [14, 15]. Furthermore, it is estimated that South Africa has between 200 000 and 350 000 registered THPs, including herbalists [16]. They are geographically, economically and culturally accessible [17].

According to SA Info, South Africa’s health-care services are available to approximately 80% of the population [18]. The doctor-patient ratio is projected to be 0.77 per 1 000 in the country. Most (73%) of the medical doctors in South Africa work in the private sphere leaving only one doctor for every 4 219 people in public hospitals. In contrast, the ratio of THPs to the general public is 1:500 [17]. THPs therefore fill an important gap in the primary health-care market [19], though in this study, they often relied on medical diagnosis before treating patients.

The South African Traditional health Practitioners Act (22 of 2007) recognises diviners (*amagrirha: isangoma*), herbalists (*inyangas*), traditional birth attendants and traditional surgeons [20]. Philander [21] found that people also readily consult *bossies- or kruiedoktors* (Bush doctors) and Rastafarians, who fill a ‘gap’ in the health-care system and help to cope with the demands of an increased disease burden in their local communities. In South Africa’s policy documents (such as the Draft policy on African traditional medicine no 31271 of 2008 [22]), THPs are represented as the custodians of local knowledge about medicinal plants. According to Thornton, there are five domains of healing practice in South Africa; divination, herbs, placating or control of ancestral spirits, the cult of foreign *ndzawe* (foreign) spirits, drumming and dancing [23].

Recently, there has been increased interest in the attitudes, beliefs, knowledge, practices and the role that THPs play in treating infectious diseases, especially HIV/AIDS and TB [17, 19, 24–28]. However, many of these studies are generic and they focus largely on the role of the THPs in primary health care. Little is known about how these THPs conceptualise infectious diseases or about the plants they use to manage HIV and OIs in South Africa. Therefore, this study focuses on how diviners and herbalists, who are consulted for cases of HIV and OIs, conceptualise, manage and treat these diseases using traditional herbal medicines. Through an ethnobotanical study, this study aimed to fill this knowledge gap. In this article, we refer to diviners and herbalists as THPs [20] except where a distinction is made between the two.

## Methods

### Study design and sample size

The study utilised mixed methods to explore the conceptualisations of HIV and associated OIs, and to collect and prepare voucher specimens of the medicinal plants used for treatment. This research is part of a broader study initiated by the Multi-disciplinary University Traditional Health Initiative (MUTHI) to document medicinal plants used in several African countries in the treatment of various infectious diseases.

**Figure 1:**
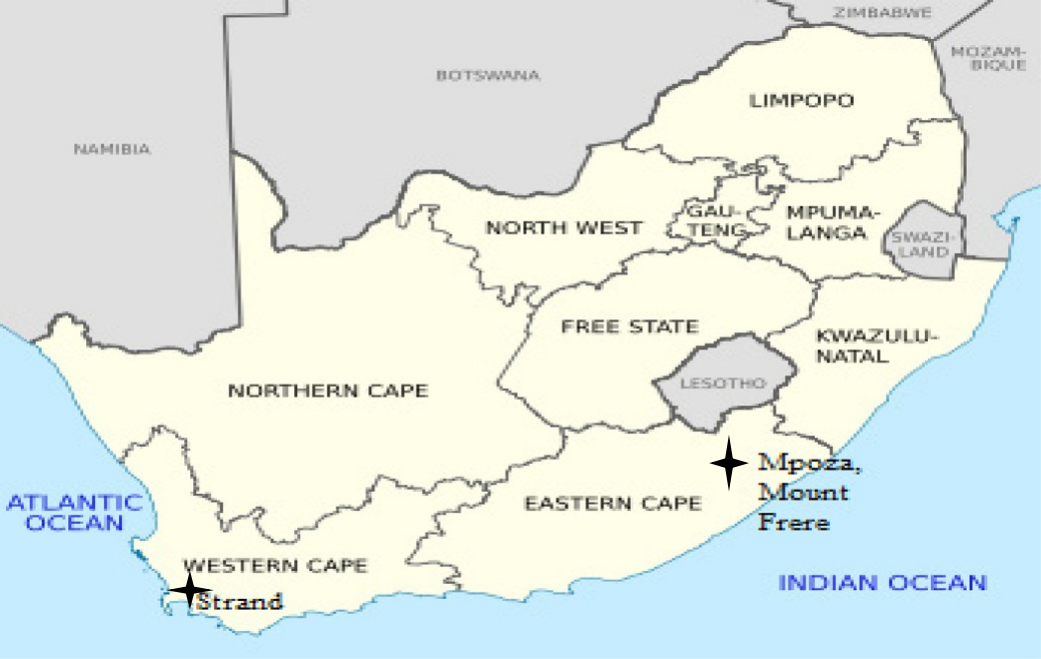
**Study sites: Strand, Western Cape Province and Mount Frere, Eastern Cape Province, South Africa.**

Since THPs in Strand, Western Cape, collect or order medicinal plants mostly from Mount Frere, Eastern Cape (Figure 1), both areas were studied. A total of 36 diviners and 17 herbalists were sampled in both study sites. A meeting was held with the chair of the local Traditional Healers Association (THA) in each study site and prior informed consent was obtained stipulating the purpose for collection and use of medicinal plants. Interviews were conducted in the preferred language of the respondent (IsiXhosa and IsiZulu). Plants were collected in two fold and voucher specimens prepared according to the specifications of the herbarium at the University of the Western Cape. The chair of the THA at each site helped to identify and sample THPs for the focus group discussions (FGDs). The THPs interviewed, were selected through a non-probability “snowball” sample [29]. THPs were asked to identify other potential participants to participate in the study. Field work took place from January 2012 to October 2012. Selection criteria included THPs who had plant collection permits and belonged to a local THA.

### Study setting

The study was undertaken in two provinces because many of the THPs in Strand received their training in Mount Frere area (Mpoza). They also collect or “order” medicinal plants from relatives or plant gatherers in Mount Frere. Strand is an urban coastal town approximately 50 km from Cape Town, located in the Helderberg district with a total population of approximately 50 000 of which 8% is black [30]. Strand has a large area with informal housing that accounts for 16.7% of the population and unemployment rate of 25.5% [31]. Approximately 5.4% of the population is affected by HIV and TB [32]. Mpoza is a semi-rural village located in the Mount Frere district and has a total population of 7 655 of which 99% is black [33]. The average number of people per household is 7.4 [34]. Poverty and unemployment is high and is seen as contributing to the high prevalence of infectious disease [33]. Mpoza has an HIV prevalence rate of 15.2% [33].

### Demographics of participants

In our sample of THPs, 73% were diviners and 27% were herbalists. The herbalists were all males and belong to the Zionist church. The diviners were all females and do not belong to any religious denomination. The THPs’ ages ranged from 32–92 years. Their level of school education at both study sites was 58.49% who completed primary education, 30.19% secondary education, and 1.06% tertiary education. Only 2.12% had no formal education. Nearly all (98.41%) of the THPs were full-time practising diviners or herbalists and were known in the areas where they lived. THPs received most of their income from their healing practices, but also supplemented their income with informal trading of food and livestock. The remaining (1.59%) were part-time THPs and employed as domestic workers, gardeners or public administrators. All of the THPs were registered with a local THA, had plant collection permits and practiced from their homes. Table 1 shows the demographic characteristics of THPs in both study areas.

**Table 1:** **Demographic characteristics of traditional health practitioners in both study areas**

Characteristics	Traditional health practitioner n (%)
Gender	
Male	17 (32.07)
Female	36 (67.92)
Age group (years)	
30-40	19 (35.85)
41-50	6 (11.32)
51-60	11 (22.64)
61-70	7 (20.75)
71-80	5 (9.43)
81 +	5 (9.43)
Sources of information	
Ancestral	36 (67.92)
Male family member	13 (24.53)
Self-taught	4 (7.55)
Years of experience	
Less than 5	12 (22.64)
6-10	5 (9.43)
11-15	10 (18.87)
16-20	15 (28.30)
Above 21+	11 (20.75)
Experience in treating TB	
Yes	53 (100)
No	0 (0.0)
Experience in treating HIV	
Yes	6 (11.32)
No	47 (88.68)
Experience in treating OIs associated with HIV	
Yes	51 (96.23)
No	2 (3.77)
Level of education	
Primary	31 (58.49)
Secondary	16 (30.19)
Tertiary	2 (1.06)
No education	4 (2.12)

TB: tuberculosis; HIV: human immunodeficiency virus; OIs: opportunistic infections.

### Data collection

Data was collected through individual semi-structured interviews and FGDs. In both cases a purposive snowball sample technique was used to approach THPs to participate in the study. All participants selected, were practising diviners or herbalists and were known as such in either Strand or Mpoza. They were all thus key informants.

The same topic schedule (see Additional file 1) for discussion was used for each FGD. This was done to enhance consistency in the conversations that emerged between participants. For the first round of FGDs, participants were not preselected. Instead, all of the participants who were interviewed and who participated in plant collection were invited to participate. Participants were informed of the date, time and location for the FGDs. Participants were offered refreshments before the FGD began. The venues for the FGDs were familiar to all of the participants and the translator was a local and well-known among them. All of which made them feel comfortable. All participants were informed of the aims, objectives ad themes of the FGD in their preferred language (IsiXhosa or IsiZulu) and signed an informed consent document. Participants then completed a register and name tags were made for them. The first and second authors acted as facilitator and moderator respectively. Both have training and experience in managing focus group discussions. The facilitator took care to direct and redirect questions to ensure participation by all. The initial discussions were started with engaging questions such as “how long have you been practising as a THP?” and “from who did you acquire this knowledge?” Then exploratory or probing questions such as “what causes HIV?” and “what is the role of the ancestors in healing?” asked. Where necessary, probing questions were used to further explore underlying thoughts and assumptions. Finally, exit questions were used to ascertain if anything had been overlooked in the discussion. The facilitator and moderator listened attentively and gave sensitive and impartial responses to participant’s input.

After each FGD, the data were reviewed to see if new themes or information had emerged. At Mpoza, two FGDs were planned and held at the district health-care facility. Each FGD involved 13 diviners (all female) and five herbalists (all male). These THPs constituted all those practising in Mpoza at the time of interview. After the second FGD, no new information, themes or insights arose from the data and saturation had been reached [35]. In Strand, where there are larger numbers of practising THPs, the first FGD included ten diviners and six herbalists. The second group involved seven diviners and five herbalists. Twelve diviners and seven herbalists participated in the third FGD. All the FGDs were held in the Broadlands Municipality office, Strand. As in Mpoza, after each FGD, the material was transcribed and reviewed. After the third FGD, no new information or themes arose. Nevertheless, to ascertain whether there were distinctions between the ways in which diviners and herbalists understood HIV, TB and OIs, one FGD was done with diviners (15) and another with herbalists (10) only. There were no distinct differences in the information received. The information generated through the FGDs was triangulated with the in-depth interviews and current literature.

In Mpoza, the semi-structured interviews were conducted with the aid of fieldworkers who acted as translators. Interviews were held with eight diviners and two herbalists. In Strand, twenty-nine diviners and five herbalists were interviewed*.* With the permission of THPs in both study sites, the interviews and FGDs were tape recorded. Extensive field notes and photographs were taken with the consent of the THPs. Interviews were transcribed and cross-checked with interpreters and a sample of THPs for accuracy and saturation.

The collected plant specimens were dry pressed in the field. The scientific names of the plants were located by the authors who cross-checked their vernacular names and photographs with available literature. The dry pressed plants were then taken to the herbarium at the University of the Western Cape. Several botanists at the University herbarium examined the plant pressings together with collection notes such as flora, vernacular name, locality, GPS coordinates, habitat, plant distribution when collected, plant parts used, preparation methods, administration methods and a comprehensive plant description against the herbariums’ existing database of voucher specimens. The botanist identified the plants and their names were updated by the authors using the Kew’s plant list “http://www.theplantlist.org/”. A complete set of voucher specimens were then deposited at the herbarium at the University of the Western Cape for future reference.

### Data analysis

After the interviews and FGDs, all information was carefully scrutinised and the first open codes were linked to sets of data. When no new information was generated during data collection and analysis and codes were not changed anymore, saturation had been achieved. Such codes included, *ityhefu* (poison), dirt, *ungcoliseko* (pollution-environmental)*, idliso* (pollution-spiritual), and witchcraft and would cohere under the theme HIV causation (emic). The prevalence of themes was determined by the number of sections of research material that were encapsulated by related codes [36].

In this way, data were analysed using a thematic content analysis approach [37]. The data underwent several stages of analyses starting with initial transcriptions and scrutiny of the material to check for coherence and saturation. Data from different respondents were compared with each other to identify patterns and themes that recurred or were common [38]. Thereafter, the transcriptions were presented to a random sample of participants in a respondent-feedback session for cross-referencing and editing where necessary. Finally, the themes were then discussed with colleagues to scrutinise its reliability and validity and to substantiate that the results were not anecdotal.

### Ethical considerations

Ethical approval for the overall MUTHI project was obtained from the Senate Ethics Committee UWC. Prior informed consent was sought from the THA and village head in Mpoza and the chair of the THA in Strand. Prior informed consent was also obtained from each participant. Interviews were conducted in the preferred language of the respondent (IsiXhosa and IsiZulu).

## Results

This study found that among the 53 THPs interviewed, 11% had treated clients who had been diagnosed as HIV+ by a qualified health-care worker in a clinic. Ninety six per cent of the THPs treated, but did not diagnose, clients whom they suspected had HIV. These people had thus not been biomedically diagnosed. Instead, the THPs based their assumption that a client probably had HIV on a combination of physical symptoms such as weight loss, continued feverishness, constant tiredness, diarrhoea, swelling in the armpits and stomach as well as red “blister-like” sores (*ukugubhuka:* “belt”) on the neck, waist and arms or thrush (*amaqhakuva asemlonyeni*) in and around the mouth or genitalia. THPs from Strand and Mpoza reported that they had only treated five (9.4%) cases of TB which had been diagnosed in the biomedical health-care system. However, 67% of THPs treated clients presenting with symptoms of TB, namely chronic cough, wasting, sweating, headaches and chest pains. Furthermore, THPs also relied on past experience with treating such symptoms and guidance from the ancestors.

### Perceptions of HIV and associated opportunistic infections

**Figure 2:**
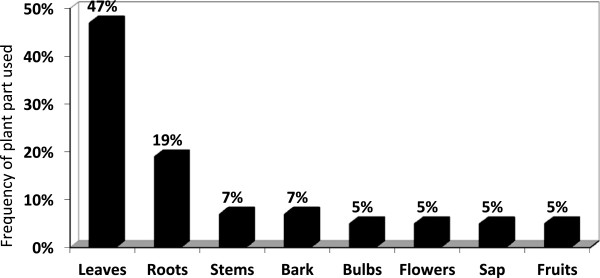
**Traditional Herbal Practitioners’ (THPs) conceptualisation of disease.**

**Figure 3:**
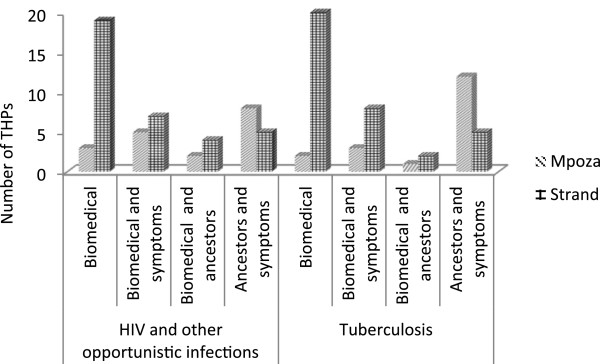
**Traditional Herbal Practitioners’ conceptualisation of the aetiology/cause of disease.**

None of the THPs in both study areas claimed to have diagnosed HIV or TB. THPs always relied on a diagnosis of a qualified health-care practitioner from a local clinic. THPs in both areas had homogenous ideas about how to treat the symptoms of OIs, which is not surprising, since many of the THPs in Strand originated from or had received their training in the Eastern Cape. Practices varied between diviners and herbalists. Diviners relied more on guidance from the ancestors when treating OIs, while herbalists generally practice with herbs only, and in some instances would consult a diviner when a client presented with an uncommon symptom. Drawing on responses from semi-structured interviews and FGDs, THPs’ conceptualisation about HIV, and especially OIs, fell across four spheres of influence as can be seen in Figures 2 and 3. While it was evident that THPs relied on a biomedical diagnosis, they also understood the symptoms with which a client presented as a combination of biomedical diagnosis and symptoms, biomedical diagnosis and ancestors; and ancestors and symptoms. These ideas about HIV and OIs are also related to THPs’ understandings of the cause of disease which were further grouped into three categories, i.e. physical, environmental and spiritual pollution. These categories were sometimes used interchangeably as causes of disease and varied among the THPs and between THPs from Strand and Mpoza (see Figures 2 and 3).

Most THPs from Strand had some knowledge about the physical causes of HIV, as well as the symptoms of OIs, such as red “blister-like” sores and thrush, which were the most frequently reported. In Strand, THPs had exposure to HIV education and showed a high level of biomedical knowledge of the symptoms and treatment of HIV and associated OIs. Many of these THPs (87%) had attended at least one workshop or an awareness programme around HIV, TB and OIs. They also had access to information about these diseases through public libraries, clinics and the information available in the media. In Mpoza, general knowledge of the physical causes and symptoms of TB was moderate, however, they were less knowledgeable about HIV. When these THPs treated clients with symptoms such as swelling in the armpits and neck, red *ukugubhuka* (blisters with fluid) on the arms, neck, around the waist (“belt”) or with *amaqhakuva asemlonyeni* (mouth sores) in or around the mouth and/or the genitalia, they suspected HIV infection. Overall, the THPs firstly relied on medical diagnoses. They also looked for physical symptoms such as rashes, swollen lymph nodes, mouth and genital ulcers, vaginal discharge, weight loss, diarrhoea etcetera. TB was suspected when clients had persistent coughing, bloody sputum, weight loss and on-going feverishness. THPs were simultaneously guided by their ancestors in diagnosing a client’s ailment and for treatment options. Treatment methods varied from THP to THP.

As seen above, the perceptions of THPs pertaining to disease and treatment incorporate biomedical diagnoses, physical symptoms, ancestral guidance, as well as the possible negative influences of witchcraft or pollution. Ultimately, for THPs, healing is about addressing underlying states of imbalance caused by poor physical environment, disturbed relationships with the ancestors, negative relations between people (witchcraft) and with the spiritual world (pollution).

### Physical, environmental and spiritual causation

Twenty-four per cent of the THPs from Mpoza and 63% from Strand, understood HIV as a disease which is spread through blood and through physical intercourse. HIV transmission occurs through contact with “dirty” or “poisonous” blood e.g. by using contaminated blades to do circumcisions, semen, vaginal fluids, and intercourse with a menstruating woman.

HIV can also be caused by environmental pollution (*ungcoliseko).* This “pollution” occurs when people become ill due to contamination, smog, exposure to sick people and seasonal changes. In Mpoza, the THPs furthermore linked the coming of HIV to modernization and urbanisation. This was exacerbated by the building of the N2 highway which drew “foreigners”, truckers and passers-through who bring their infections from the “outside” to the rural areas.

Spiritual “pollution” or (*idliso-* poison*)* can also result in HIV. *Idliso* is caused by the neglect of the ancestors and necessary rituals and manifests in poor social behaviour, promiscuity, adultery, drinking too much and smoking. Urbanisation was reported as the biggest threat to traditional values and ways of living. THPs suggested that in the past, their ancestors lived as agriculturalists. According to Mr Y from Mpoza:

‘…*they were healthy and fit. My parents were old when they died. There was no fatty food and cars to drive. We walked. These people came and brought the HIV. When my father taught me the herbs, I did not know about HIV. It came when the men left their wives and daughters to work in far places. They leave on trucks on that road and send money home. After a while, what happens? They found other women. The girls start selling themselves along that road. The mothers find other men. You have broken families*’.

In Strand, THPs reported that sexual intercourse with more than one sexual partner, drinking too much, prostitution and even migration lead to promiscuity and adultery and are examples of both physical and spiritual “pollution.” Strand THPs reported that young women and girls are especially vulnerable; because of poverty they drop out of school, but cannot find employment and spend time at *shebeens* (informal drinking places), where they find random sexual partners and are exposed to drugs. According to Mrs D from Strand:

‘*They [young women and girls] are easily seduced by businessmen who “come into the village with their fancy big cars and pick up the girls’*

In another interview, Mr S, from Strand reported:

‘*They go to the shebeens on Friday and Saturday nights. Those places are bad because they drink and shoot pool and that is where they pick up the young girls. The children, they bunk school to go there*’.

As seen above, the behaviour of, especially younger people, is linked to poverty, unemployment, migration and the breaking down of traditional ways of living. According to Dickinson [39], THPs are seen as custodians of traditional values and social order. This was found to be the case in Mpoza and Strand where THPs were actively involved with self-organised awareness programmes about sexuality.

Nearly all of the THPs in Mpoza and Strand (92%) attributed TB to exposure to people infected with TB, coughing and sputum. Vulnerability to TB was reportedly heightened because of smoking, poverty and air pollution. In Strand, TB was commonly seen as deriving from “dirtiness”, poor housing, economic circumstances, poor nutrition, drinking too much, smoking and low resistance. TB infection settles in the chest and results in heavy coughing, night sweats and chest pain. TB may also be caused by the sharing of utensils, poor hygiene and coughing over people. THPs furthermore associated TB with HIV infection.

Both TB and HIV can reportedly be the result of witchcraft. People who neglect ancestral rituals can be spiritually “polluted” at crossroads, by strangers or malevolent individuals who wish to harm them and who bring *idliso* and the possibility of witchcraft. Mr B from Strand stated:

*‘…so when a person who has TB spits in the road, that spit…the wind blows it and it makes other people sick with this TB when they breathe it…*’

THPs most frequently used a biomedically informed explanation of TB, but they did not exclude *idliso* and witchcraft as causes. They accepted that their clients would visit health-care facilities and in most cases encouraged clients to be tested before treating them. There is thus a diversity of ideas about causation which overlap and may best be understood on a sliding scale from naturalistic to “personalistic” causes [40, 41]. Thus, in certain cases, THPs also see witchcraft and “spiritual pollution” as causal.

There is an aetiological connection between biomedical explanations, the impact of poverty, urbanisation, the breakdown of traditional values and the overarching role of the ancestors. According to Dickinson [39] much of THPs understandings of HIV are in line with scientific explanatory models, but there are also critical differences and competing explanations of HIV and TB. Thus, biomedical and local explanatory models intermix and are not easily disentangled.

### Treatment process

THPs follow diverse procedures in treating their patients. The scope of their practice is wide-ranging: from the collection and preparation of herbal remedies to counselling, sucking of blood from wounds, cleansing ceremonies, dealing with births, circumcision and using a range of physical and spiritual techniques. Most THPs reported that HIV was a relatively new, incurable disease and they are not trained to deal with it. Red “blister-like” sores on the neck, waist and arms or thrush on the genitalia or mouth region on the other hand were common and frequently treated. The treatment process varied between THPs. They consulted the ancestors about plants to treat their clients and performed rituals during treatment. Moreover, 89% of THPs encouraged their clients to consume TM together with ARV’s and TB medication which are available from the local clinic.

The treatment process for HIV, TB and OIs involves three stages of regenerative work depending on the symptoms and severity. These stages implement various treatments to address the body from inside out. For THPs from Mpoza and Strand, treating a client is about reinstating a state of balance which was disrupted by social behaviour, *idliso* or witchcraft. The healing process is consequently about expulsion of *idliso* “poisons”. Many of the THPs in Mpoza and Strand believe that the healing process involves a systematic support of the body’s own ability to heal itself as opposed to relieving immediate symptoms.

### Treatment pathway

The first stage of treatment happens at the ancestral shrine and is aimed at cultivating strength and getting the person back on his/her feet. A THP will prepare nutritious food to “cleanse” the client from the “inside”. The client is also given a weak infusion of a plant remedy (singly or as a mixture of plants). At this stage of treatment, the client is not well enough for a strong dose of the remedy. Pregnant woman, children and infants are usually given a weaker dosage.

The initial treatment also involves purging. The plants used to treat the client are meant to make him/her sweat, throw up, urinate or excrete the *idliso* from the body. OIs such as red sores, thrush, rashes and other symptoms are treated with plant mixtures. In the case of TB, the chest of the client is massaged with an ointment or lotion to give relief. The first stage of treatment typically lasts a day or two.

In the second stage of the treatment process, the client is well enough to leave the THPs shrine. He/she is given a remedy with instructions concerning the dosages. In the case of HIV and TB, the medicine is usually taken three times a day: in the morning, in the afternoon and in the evening after meals. OIs are treated with a topical herbal remedy administered thrice a day or when experiencing discomfort. This stage also includes a stronger plant remedy. The second stage of treatment spans over two weeks.

The third and final stage of treatment usually lasts four weeks or more. The most potent plant remedies are now administered. Throughout the process, the client visits the THP in follow-up sessions to examine progress. Most THPs in Mpoza and Strand reported that the symptoms of HIV and TB disappear after six weeks of successful treatment. A few THPs argued that the plants they use are “strong” and the symptoms may dissipate after a client had been treated for a week or two. Generally, treatment lasts until the symptoms disappear.

One of the most common reasons for HIV infected clients to visit THPs was when ARV’s have side effects such as throwing up, stomach cramps and headaches. THPs then provided a mixture of plants to be taken after the consumption of ARV’s to stop the reported side effects.

### Preparation

According to the diviners from Mpoza and Strand, the ancestors inform them when a client will visit them. The ancestors communicate through dreams, but may also utilise beads, visions, animals and weather to connect with the living. Diviners perform rituals to communicate with the ancestors about the specific plants needed to treat individual clients. Herbalists*,* on the other hand, generally rely on their existing knowledge, the physical symptoms a client presents with or medical diagnoses before treatment. Plants are collected for treatment from the field/bush. In Strand, THPs make use of the plants available to them (fresh or dried), but prefer to ‘order’ specific plants from family members or plant gatherers in Mpoza.

Plants deemed especially powerful in treating HIV and OIs are collected and dried under the guidance of ancestors. These plants may be specific for HIV or TB or are used for individual clients. According to the THPs from Mpoza and Strand, no two clients are treated in the same way.

The plants (whole or in part) are dug up. The remaining roots are covered to preserve whatever is left behind for sustainability and as a sign of respect to the ancestors and to the land. This reportedly ensures the continued growth of the plant for future use. In their shrines, THPs set about grinding or grating the plants, wholly or in part, depending on the intended application, for example as a tea, syrup, a paste applied to the skin or a powder. The plants are then stored in plastic or glass containers for later use.

### Plants used

**Figure 4:**
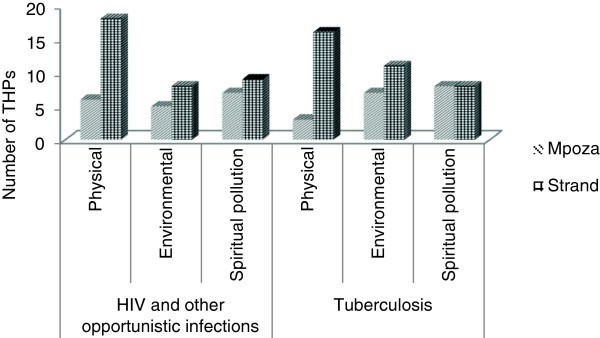
**Plant parts used by traditional health practitioners for remedies to manage HIV and commonly associated opportunistic infections.** (N = 17 plants).

THPs identified 17 plants belonging to 12 families used in the management of suspected HIV and treatment of OIs. These plants are indigenous to South Africa. Table 2 gives a list of the plants used to manage HIV and to treat OIs. The most frequently reported plants were *Hypoxis hemerocallidea* (48%), *Asparagus densiflorus* (27%) and *Lessertia frutescens* (L.; 25%). Figure 4 indicates the frequency of use of the different plant parts used by THPs to prepare remedies to manage and or treat HIV and OIs.

**Table 2:** **Plants used to manage symptoms of HIV and opportunistic infections**

Scientific name; (author); [voucher number]	Family	Common/Local names	Plant part used	Uses	Notes
Agathosma apiculata E.Mey. ex Bartl. & H.L.Wendl. [TB/DD/7]	Rutaceae	Honey buchu (Eng); *Umbhucu* (Zulu)	Leaves	The leaves are crushed into a pulp and applied directly to the skin or mixed with Vaseline.	Used externally to treat shingles and other skin infections, such as sores around the mouth and genitals.
*Alepidea capensis* (P.J.Bergius) R.A.Dyer [TB/DD/1]	Apiaceae	Large tinsel flower; *Iqwili* (Zulu)	Roots and stem	A decoction of dried roots (a few dried roots boiled in 200 ml water) is used for abdominal and chest pain associated with HIV and TB. Dried stems and roots are smoked, or powdered and taken as snuff.	Used as incense to communicate with the ancestors. Inhaling the smoke can result in hallucinogenic sensations and “bad” dreams.
*Aloe ferox* Mill [TB/DD/16]	*Asparagaceae*	Bitter aloe, Red aloe; *ikhalana, ikhala, intelezi* (Zulu)	Leaves	The leaves are cut into cubes and dried. They form black crystals which are consumed as a purgative and immune booster. The sap of Aloe is applied to skin for shingles and thrush.	Used for HIV and TB
*Bulbine alooides* (L.) Willd. [TB/DD/15]	*Xanthorrhoeaceae*	*Rooiwater* (Red water)	Sap	The sap is used internally as a purgative and externally, applied directly to the skin, to treat shingles and thrush.	THPs reported that it is a blood purifier attributable to the reddish colour of the sap.
*Mentha longifolia* (L.) L. [TB/DD/9]	*Lamiaceae*	*Imboya* (Zulu)	Leaves	Used in a remedy to combat abdominal pains and as a purgative. THPs mash the oily leaves into a pulp and apply to the skin for shingles and herpes zoster	The plant is used in moderation. This was because traditional healers reported that, in the past, some of their livestock died from consuming the plant.
*Cussonia paniculata* Eckl. & Zeyh [TB/DD/4]	Araliaceae	Mountain cabbage tree; *Umsenge* (Zulu)	Leaves and bark	The leaves are used to combat indigestion and as a purgative. The leaves can be chewed straight from the tree or used in a decoction. The bark and leaves are utilised together on the skin to treat shingles or drank as part of a remedy to treat HIV symptoms.	This is also given as a general immune booster and tonic. THPs also reported that they use the roots of the plant as a treatment for malaria.
*Cussonia spicata Thunb.* [TB/DD/14]	Araliaceae	Wild cabbage tree *Intsenge* (Zulu)	Flowers, roots, fruit and stems	The flowers, roots, fruit and stems are used to treat shingles by directly applying to the skin. Also used as a purgative, a general tonic and an immune booster.	*The flowers and roots are used* to treat malaria, stomach complaints and venereal disease. The fruits are eaten raw or in a decoction.
*Helichrysum herbaceum* (Andrews) Sweet [TB/DD/8]	*Compositae*	Natal guarri, Natal ebony or large-leaved guarri; *Umtshekesane* (Zulu)	Roots	Used to treat pain and fever, stomach ‘complaints’, worms and chest complaints associated with TB. For respiratory problems (such as TB) the roots are pulverized, boiled and used in a mixture.	The roots and bark are used as an ingredient in a variety of remedies.
*Foeniculum vulgare* Mill. [TB/DD/2]	Apiaceae	Fennel; *Imboziso* (Zulu)	Leaves	THPs use it as a diuretic, anti-spasmodic and calmative herb. The plant is utilised internally and externally in decoctions (50 g of fennel leaves to 500 ml water) to treat genital thrush as well as thrush of the mouth in cases of HIV infection.	Frequently used to treat TB. Administered to the chest and given as a warm brew in a remedy to treat coughs, chest pains and inflammation.
*Leonotis leonurus* (L.) R.Br. [TB/DD/10]	*Lamiaceae*	March flower; *Hlodlwana* (Zulu)	Leaves	Fresh leaves are applied as a dressing to septic ulcers and sores. The bulb is sliced and boiled in a remedy as a diuretic. The stem and flowers are boiled with water and drank as syrup.	Used in a remedy for chest complaints associated with TB. A pulp of the same parts is applied to the abdomen of a woman who is struggling to conceive. This later treatment includes prayers to the ancestors, as well as other rituals such as burning dried *imphepho* over the abdomen of the woman.
*Hypoxis hemerocallidea* Fisch., C.A.Mey. & Avé-Lall. [TB/DD/5]	Hypoxidaceae	African potato; *Inkomfe* (Zulu)	Roots	The dried root is grated and added to a remedy for HIV symptoms. Also used to treat TB, internal cancers, malaria, and heart diseases.	The plant is highly regarded among THPs to treat HIV.
*Lessertia frutescens* (L.) Goldblatt & J.C. Manning [TB/DD/6]	*Leguminosae*	*Kankerbossie; uNwele* (Zulu)	Leaves	The leaves are used in a remedy to treat HIV as well as for HIV-TB co-infection. The plant is also used to treat people with suspected cancer.	THPs use it as a blood-purifier, an all-purpose tonic, anti-depressant and for respiratory conditions associated with TB such as asthma, bronchitis, influenza, wasting and bronchitis.
*Lippia javanica* (Burm.f.) Spreng. [TB/DD/11]	Verbenaceae	Fever tea/ Lemon bush; *Uvevane* (Zulu)	Leaves	Applied singly to the skin or mixed with Vaseline to make an ointment. Drank as a tea (approximately 50 g added to a cup of boiling water) for the treatment of coughs, colds and bronchial problems.	Effective for pain, fever, malaria, influenza, measles, and for lung infections. Is also used as an insect repellent and in cleansing ceremonies.
*Prunus africana* (Hook. f.) Kalkman [TB/DD/12]	Rosaceae	*Rooistinkhout* (Afr.), *Inyazangoma-elimnyama* (Zulu); *uMkakase* (Xhosa)	Bark	The bark is ground and used in a powdered form in a remedy to treat HIV and TB co-infection and chest complaints.	The tree is reportedly poisonous when raw and used in moderation.
*Merwilla plumbea* (Lindl.) Speta [TB/DD/13]	*Asparagaceae*	Wild squill, Blue squill, Blue hyacinth, *Blouberglelie* (Blue mountain Lillie), and *Blouslangkop* (Blue snake head) *Inguduza* (Zulu)	Bulbs	The bulbs are used fresh or dried, warmed or even burnt and administered externally to treat shingles in clients with suspected HIV. The lobes of the bulbs are used in a decoction (a few lobes added to 100 ml of boiling water) to treat shingles.	The plant is considered highly toxic when raw and should be handled with extreme caution.
*Urtica dioica* L. [TB/DD/3]	Urticaceae	Stinging nettle; *Umbabazane* (Zulu)	Leaves and roots	The leaves and roots are boiled with other plants as an anti-inflammatory remedy.	Eradicates “poison” in the body as part of remedy to treat HIV and TB co-infection. Used as a calmative and as a purgative. Reportedly used to treat people with attention difficulties and apathy.
*Asparagus densiflorus* (Kunth) Jessop [TB/DD/17]	*Asparagaceae*	*uNwele* (Zulu)	Leaves	An infusion of the plants leaves is used to treat abdominal pain, as a general tonic and immune booster and as a cleansing agent to rid the body of “poison.” Also used to treat thrush and ulcers in the mouth associated with HIV.	THPs reported that *uNwele* is one of the “strongest” plants used for HIV.

## Discussion

Among THPs in both study sites, there is a diversity of explanations of the causation of HIV and TB. In the seminal literature on disease and illness aetiologies, differentiations are often made between biomedical and ‘traditional’ systems of healing [40, 42–45]. In relation to the latter, Foster [40], argued that, aetiological explanations can shift between “naturalistic” and “personalistic” causes. Diseases, in turn, are “biomedical” conditions [45] or “pathological states” and are culturally recognized as such or not [44]. Illness is broadly seen as a “sociocultural category” [45]. It involves peoples’ own understanding and experiences of particular “socially disvalued states” and can include disease [42]. In our study, the simultaneity of observed and magical/supernatural features in TM is acknowledged. Furthermore, we found that THPs’ aetiologies are informed by biomedical understandings, but are intertwined with personalistic ideas about causation, such as environmental and spiritual pollution which includes witchcraft.

In Mpoza, perceived causal factors lay more towards personalistic explanations, probably because THPs were less exposed to HIV and TB education. THPs regarded HIV as a relatively new disease. This was also found among THPs in other studies in rural South Africa [26, 39]. Dickinson [39] observed that THPs have no shared source of knowledge upon which other THPs can draw. THPs therefore appeared to concentrate on the treatment of symptoms. Clients also often consult THPs once symptoms of OIs, such as red sores and thrush, occur.

THPs in Strand generally depend on biomedical diagnosis before treating a client. THPs from Strand also reported working with allopathic doctors and local clinics to refer cases of suspected HIV or TB for diagnosis. This occurrence was also found among THPs in other studies in South Africa [10, 27, 46]. In this study, THPs were aware of and generally accepted that their clients will seek different forms of health-care services and sometimes several simultaneously. Even with the knowledge that a client is on ARV or TB treatment, THPs encourage the utilisation of TM. This was frequently the case when clients visited THPs for adverse side effects of ARV or TB treatment. Recent studies have reported the trend of co-utilization of herbal remedies and ARV’s for people living with HIV/AIDs [6, 7].

THPs’ conceptualisations of disease varied and our study observed that 4% of the total sample of them believed that they could cure HIV. This has also been reported in another South African study [46]. Most THPs, however, stressed that HIV is incurable and that herbal remedies can only manage its symptoms. HIV and TB, according to THPs, is exacerbated by poverty, inequality, poor living conditions, overcrowding, proximity to infected people, unsanitary living conditions and change in seasons and spiritual pollution.

The connection between HIV and witchcraft was made by THPs. They suspected witchcraft when no other known causes were discernable or when a client suspected that s(h)e had been bewitched. In literature, it is argued that witchcraft as a cause of HIV could be a way for people to ‘make sense’ of unexplained sickness [47–52]. In such cases, THPs sought to reconcile with the ancestors on behalf of their clients through ritual sacrifice and cleansing ceremonies. For the most part, THPs’ conceptualisations were heterogeneous and were informed by past experience, beliefs, the guidance of ancestors and physical symptoms. Thus, THPs’ conceptualisations were interwoven with medical, local and cultural understandings and were not easily disentangled. In this regard, Glick [53] argues that “it is common knowledge that in many cultures, ideas and practices are for the most part inseparable from the domain of religious beliefs and practices.” However, THPs’ conceptualisations were not grounded in essentialist understandings of disease, but were found to be constantly shifting and adapting over time and space.

This was especially evident when examining THPs’ use of plants. THPs use different plants to manage the same symptoms. When the same plants are used, the quantities and dosages vary. Diviners have different beliefs about the strength of dried and fresh plants. By contrast, herbalists have a more standardised way of “working” with plants. Herbalists are trained from youth and through an extensive apprenticeship by a male family member. In their training, they acquire knowledge about when and where to collect plants, parts to use, mode of preparation and toxicity. Herbalists reported that approximately 50% of the collected plants in this study may have adverse side effects such as nausea, vomiting and diarrhoea if administered incorrectly. Herbalists, unlike diviners, do not communicate with ancestors for guidance when treating clients. They rely mostly on medical diagnoses and symptoms. All of the herbalists also pray to God and use holy water, while administering medicinal remedies.

Generally, there was no conflict between THPs about clients, but inter-healer competition was reported. THPs also have referral networks amongst themselves, especially between those who live in close proximity to one another. THPs nonetheless reported that they do not have the same ancestral guardians and the dissemination of knowledge can only be done with the necessary rituals and goodwill of specific ancestors. THPs that have efficacious treatments tend to attract a strong client base.

Furthermore, the results of this study demonstrate that the herbal treatments administered were mostly multi-plant remedies. This is important given the possibility of resistance to single plant use in the case of HIV and OIs. Resistance to the use of multiple plant components is less likely to occur.

Several studies have been done to investigate the claims of THPs about plant remedies and to validate the efficacy of these for the management of HIV and TB [39, 54, 55]. Four of the mentioned plants: *A. apiculata*, *A. ferox*, *B. alooides*, *H. hemerocallidea* and *L. frutescens* are known to have anti-HIV properties and are used as treatment for TB by THPs [56–62]. Furthermore, in vivo efficacy and safety studies have been carried out on *L. frutescens* and *H. hemerocallidea*[59, 60]. Despite the popularity of their use and the support of the Department of Health and NGOs in some regions of South Africa, no clinical trials exist on the efficacy and safety of most of the plants collected. Moreover, there is a dearth of knowledge on the potential for herb-drug interactions with antiretroviral drugs.

## Conclusions

Seventeen plants belonging to 12 families were documented and reportedly used as ethnomedicine for HIV and TB, herpes zoster and candidiasis. Among the most commonly reported were *H. hemerocallidea L. frutescens* and *A. densiflorus.* These plants were utilised in remedies of up to five plants to treat the symptoms of suspected HIV and associated OIs. THPs generally rely on medical diagnosis before treating a client. *Idliso* (poison/pollution) is also seen as causal to especially HIV infection and is acquired through exposure to environmental and/or spiritual “pollution”. THPs also accept guidance from the ancestors as to the specific diagnosis and plants to use, when to harvest and how to administer these. The results of this study indicate that many of those afflicted with HIV and associated OIs readily consult THPs and use medicinal plants despite easy access to biomedical healthcare. It also suggests that both THPs and medicinal plants play an important role in filling a gap in public health care both in South Africa and beyond its borders. The hybridisation of medical practice in South Africa makes adherence to one or the other form of treatment difficult and confirm concerns around safety and efficacy of plant medicines. Further scientific examination of plants used to manage HIV and treat OIs should be validated for their safety and efficacy. Furthermore, health-care providers should be aware of the pluralistic treatment patients with these diseases undergo to prevent the potential of herb-drug interaction.

## Electronic supplementary material

Additional file 1: **Traditional health practitioners’ perceptions, herbal treatment and management of HIV and related opportunistic infections.** (DOCX 20 KB)

## Abbreviations

THPs: Traditional health practitioners
HIV: Human immunodeficiency virus
AIDS: Acquired immune deficiency syndrome
TB: Tuberculosis
ARV: Antiretroviral
THA: Traditional healers association
MUTHI: Multidisciplinary University health initiative
OIs: Opportunistic infections.
