# Supplementary material for: Traditional health practitioners’ perceptions, herbal treatment and management of HIV and related opportunistic infections
Source: J Ethnobiol Ethnomed. 2014 Dec 5;10:77. doi: 10.1186/1746-4269-10-77 (PMC4414384; doi:10.1186/1746-4269-10-77)
Supplement: Supplementary file 1 — Additional file 1: Traditional health practitioners’ perceptions, herbal treatment and management of HIV and related opportunistic infections. (DOCX 20 KB) [file 13002_2014_464_MOESM1_ESM.docx]

**Traditional health practitioners’ perceptions, herbal treatment and management of HIV and related opportunistic infections.**

**Focus Group Discussion topics:**

1. Plants and their (powers)
2. Herbal preparation techniques
3. Healing approach
4. Role of the ancestor in healing
5. Local use
6. Meaning of HIV, TB and Opportunistic infections (HIV, TB, OIs)
7. Cause of HIV, TB and OIs
8. Knowledge of plants’ medicinal values (who taught)
9. Source of knowledge (training)
10. Interface with biomedical healthcare

**Interview topic list regarding medicinal plants used in the management and treatment of HIV and opportunistic infections:**

1. Vernacular name of plant
2. Geographical location of plant
3. Plant part used
4. Is the plant grown or collected from the veld
5. Availability of the plant in the wild
6. Frequency of plant collection
7. Method of plant collection
8. Plant storage
9. Type of disease(s) treated with the plant
10. Method of plant preparation
11. Dosage of prepared herbal remedy
12. Other medicinal uses of the plant

**Semi-structured interview questions**

1. How long have you been practicing?
2. Have you treated clients with HIV?
3. Have you treated clients with TB?
4. What are the symptoms of HIV?
5. What are the symptoms of TB?
6. What do you think causes HIV?
7. What do you think causes TB?
8. Do you sometimes refer clients to the clinic?
9. Do you work with other traditional healers or herbalists?
